# Sulfite hypersensitivity – retrospective study in a Food Allergy Unit

**DOI:** 10.1186/2045-7022-5-S3-P65

**Published:** 2015-03-30

**Authors:** Diana Silva, Fabrícia Carolino, Ana Margarida Pereira, Natacha Santos, Alice Coimbra, José Luís Plácido

**Affiliations:** 1 Serviço de Imunoalergologia, Centro Hospitalar São João, Porto, Portugal

## Background

Sulfite hypersensitivity (SH) may have different clinical presentations, ranging from mild cutaneous symptoms to anaphylaxis. Our aim was to evaluate the prevalence and clinical characteristics of individuals with suspected sulfite allergy in our Food Allergy Unit (FAU).

## Methods

Clinical files of the 335 patients referred to the FAU of a Portuguese University Hospital for suspected food allergic reactions between January 2010 and April 2014 were retrospectively reviewed; those with suspicion of SH were selected. Patients’ demographics, medical history and SH diagnostic procedures reviewed. The allergy work-up was considered complete when an open food challenge (OFC) with 390mg of sodium metabisulfite (Bial Aristegui^®^ capsules) was performed.

## Results

Overall, 9% (n=31) of the patients were referred due to suspected SH. They had a median (inter quartile range) age of 32 (23;45) years, 52% were female, 42% atopic, 29% had asthma, 29% rhinitis and 16% chronic urticaria. Involved foods (accounting for a total of 53 reactions) were: wine (19 reactions); canned food (11); seafood (8); bottled soft drinks (7) and processed food (e.g.: pizza (5), delicatessen meats (3)). The most common manifestations were urticaria (84%) and dyspnea (36%); 3 individuals reported anaphylaxis. Patients with asthma had a higher proportion of respiratory symptoms (p=0.020). All except 4 individuals completed the study with OFC. Twenty-five (93%) had negative OFC (1 had a mild adverse event (emesis) not consistent with the previous reactions). Two (7%) women with a history of urticaria and angioedema (one with concomitant dyspnea) after wine and bottled beverage ingestion had positive OFC (both at a cumulative dose of 190mg); they presented facial and oral pruritus and urticaria; the one with previous respiratory complaints also presented coughing and emesis.

## Conclusions

In our sample, the prevalence of sulfite hypersensitivity suspicion was low and sulfite allergy was ruled out in the majority; only 2 patients had positive oral challenges. When sulfite hypersensitivity is considered, oral food challenge with sulfite is both a safe and valuable tool in the diagnostic work-up.

